# The complete genome sequences of *Erythroxylum coca* and *Erythroxylum novogranatense*

**DOI:** 10.56179/001c.39776

**Published:** 2022-10-29

**Authors:** Dawson White, Lyndel Meinhardt, Bryan Bailey, Stacy Pirro

**Affiliations:** 1 Grainger Bioinformatics Center, Science and Education Department, Field Museum; 2 ARS/USDA; 3 Iridian Genomes

**Keywords:** erythroxylum, genome

## Abstract

The flowering plant genus *Erythroxylum* contains approximately 300 species, including the economically and socially consequential crops called coca. We present the genome sequences of *Erythroxylum coca* and *E. novogranatense*, two cultigens produced for medicinal and quotidian use in the Andes and Amazon regions of South America, as well as the international cocaine industry. Sequencing was performed on an Illumina X-Ten platform, and reads were assembled by a *de novo* method followed by finishing via comparison with several species from the same genus. The BioProject, raw and assembled data can be accessed in GenBank for *E. coca* (PRJNA676123; JAJMLV000000000) and *E. novogranatense* (PRJNA675212; JAJKBF000000000).

## Introduction

The leaves of the coca plant have been used as a medicine and mild stimulant in South America for over 8,000 years (Plowman 1984; Dillehay et al. 2010). In more recent history, few plants have had such far reaching effects on human health and international relations (Restrepo et al. 2019). Coca crops produce the alkaloid cocaine: a natural insecticide (Nathanson et al. 1993), Western medicine’s first local anesthetic, and a controlled narcotic whose supply chains and illicit international markets have caused decades of social disaster.

Coca is classified into two species, *Erythroxylum coca* and *E. novogranatense* (Erythroxylaceae, Malpighiales), each with two taxonomic varieties. These two species are found only in cultivation, having resulted from independent origins of domestication from the wild *E. gracilipes* (White et al. 2020).

The two varieties used in this study, *E. coca* var. *ipadu* Plowman, known as Amazonian coca, and *E. novogranatense* var. *truxillense* (Rusby) Plowman, known as Trujillo coca, are regionally distinct crops. *Erythroxylum coca* var. *ipadu* is a cultivated by indigenous groups in the lowland Amazon basin of Colombia, Brazil, and Perú. *Erythroxylum novogranatense* var. *truxillense* is grown primarily in the dry valleys of northwestern Perú and is exported as a flavoring agent of Coca Cola®. These taxa have been crossed to produce improved hybrid varieties for the cocaine market, which are currently grown in southern Colombia and possibly southern Mexico (Casale, Mallette, and Jones 2014; Rodríguez Zapata 2015).

Complete genome sequences for E. *coca* var. *ipadu* and E. *novogranatense* var. *truxillense* will provide insight into the origins, evolution, and modern breeding patterns of coca crops, as well as the of the cocaine biosynthesis pathway.

## Methods

DNA from each species was provided by USDA/ARS Sustainable Perennial Crops Laboratory for use in this study.

Sequencing libraries were constructed with the Illumina TruSeq kit using standard protocols for the 2×150 bp format. Sequencing was performed on an Illumina X-Ten platform.

Raw, paired-end sequence data was trimmed of adapter sequence and low-quality regions using Trimmomatic (Bolger, Lohse, and Usadel 2014). Genome preassemblies were constructed using SPAdes (Bankevich et al. 2012), and finished with Zanfona (Kieras et al. 2021).

## Results

The results of genome assemblies are as follows:

**Table:** 

specimen	accession	genome size	N50
*E. coca* var. *ipadu*	JAJMLV000000000	584,053,830	71.4 MB
*E. novogranatense* var. *truxillense*	JAJKBF000000000	573,249,677	50.4 MB
